# How to establish a new medical school? A scoping review of the key considerations

**DOI:** 10.1007/s10459-024-10370-y

**Published:** 2024-09-04

**Authors:** Sneha Kirubakaran, Koshila Kumar, Paul Worley, Joanne Pimlott, Jennene Greenhill

**Affiliations:** 1 University of Queensland, Rockhampton, QLD Australia; 2 Academic Development, Division of Learning and Teaching, Charles Sturt University, Bathurst, NSW Australia; 3 Flinders University College of Medicine and Public Health, Adelaide, SA Australia; 4 Riverland Mallee Coorong Local Health Network, Murray Bridge, Australia; 5 School of Management, University of South Australia, Adelaide, SA Australia; 6 College of Business, Government and Law, Flinders University, Adelaide, SA Australia; 7 Faculty of Health, Southern Cross University, Gold Coast, QLD Australia

**Keywords:** New medical schools, Establishing, Establishment, Scoping review, Medical education

## Abstract

Establishing new medical schools in medically under-served regions is suggested as part of the solution to the problem of doctor shortages and maldistributions. Establishing a new medical school is, however, a complex undertaking with high financial and political stakes. Critically, the evidence-base for this significant activity has not previously been elucidated. This paper presents the first scoping review on this vitally important, yet under-researched aspect of medical education and health workforce planning. To better understand the process of new medical school establishment, this review posed two research questions: (1) What is the nature of the available literature on establishing a new medical school?; (2) What are the key factors to be considered when establishing a new medical school? Five databases and grey literature were searched in 2015 and 2021 for English-language articles, using search terms related to new medical schools and their establishment. Inclusion and exclusion criteria were based on relevance and suitability in answering the research questions. Seventy-eight articles were analysed both structurally and thematically to understand the nature of the literature and the key considerations involved. Structurally, most articles were descriptive pieces outlining personal and institutional experiences and did not make use of research methodologies nor theory. Thematically, thirteen key considerations were identified including reasons for establishment; location choices; leadership and governance; costs and funding; partnerships; staffing; student numbers; student recruitment; curriculum design and implementation; clinical training sites; buildings and facilities; information and technology resources; and accreditation. Significant gaps in the literature included how to obtain the initial permission from governing authorities and the personal costs and burnout experienced by founding leaders and staff. Although, the literature on new medical school establishment is empirically and theoretically under-developed, it is still useful and reveals a number of important considerations that could assist founding leaders and teams to maximise the outcomes and impact of their establishment efforts. Critically, the evidence-base underpinning this complex undertaking needs to be better informed by theory and research.

**Graphical abstract:**

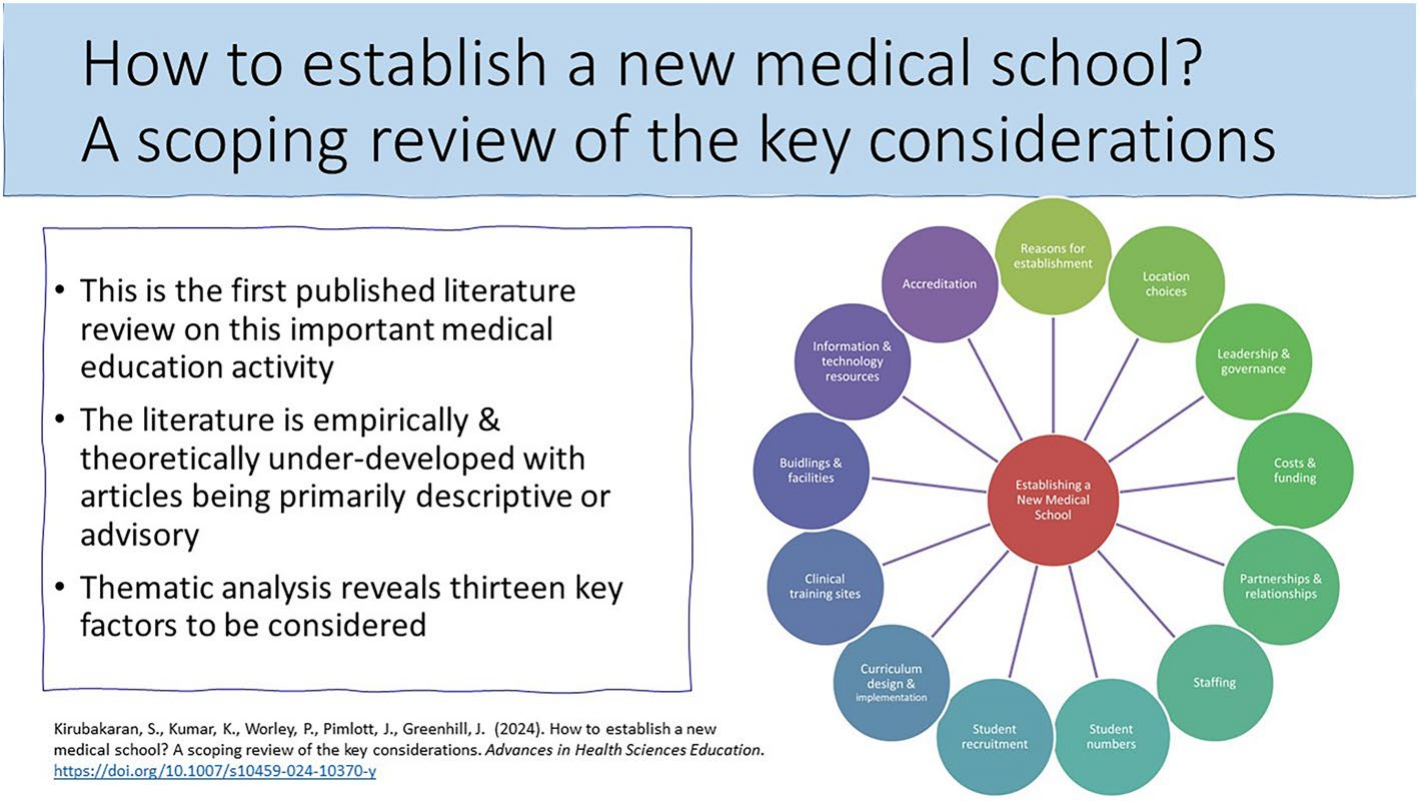

**Supplementary Information:**

The online version contains supplementary material available at 10.1007/s10459-024-10370-y.

## Introduction

Doctors are a much-needed health resource in every region, state, and nation of the world. Nonetheless, the maldistribution of doctors in regional, rural, and remote locations of both high- and low-resourced countries has been a persistent problem across the globe for many decades (Haakenstad et al., 2022; Strasser & Strasser, 2020; World Health Organization, 2018). Establishing new medical schools in medically under-served regions is suggested as part of the solution to this problem (Boelen, 2018; Boulet et al., 2007; Greenhill et al., 2015; Rourke, 2010).

Although the number of doctors practicing in a region depends on a multitude of complex socio-political factors (Beckett & Morrison, 2010; Boulet et al., 2007; Frenk et al., 2010; Murray & Craig, 2023), the presence of a medical school increases the likelihood of improved physician density (Boulet et al., 2007; PWC Consulting, 2002; Tesson et al., 2009). Even though “increasing the number of medical schools in [physician] low-density areas” (Boulet et al., 2007, p. 24) sounds like a plausible solution, establishing a new medical school is a significant undertaking involving substantial social, political, economic, educational, and organisational considerations (Australian Medical Council, 2023; Bin Abdulrahman & Saleh, 2015; Hays et al., 2019a, b; Liaison Committee on Medical Education, 2023; Whitcomb, 2018; World Federation for Medical Education, 2020). Despite the complexity, almost one hundred new medical schools are being established around the world every year (Bedoll et al., 2021; Boulet et al., 2007; Duvivier et al., 2014). Although some parts of the world are slowing down their rate of establishing new medical schools (Whitcomb, 2018), others are seeking to start more (Callan, 2022; Hicking, 2022; Moore, 2023; Step Communications, 2023; University of Surrey, 2022).

Adopting a perspective of potential founding leaders seeking to establish a new medical school to address workforce shortages/maldistributions and health inequities in medically under-served areas, we posed a series of hypothetical questions: How do founding teams go about the complex process of establishing a new medical school? What crucial factors need to be considered? What literature is available to assist them? A preliminary search of the literature identified that there were no published reviews on the *process* of establishing a new medical school. While the broad and multi-faceted scope of such a review may have been a deterrent, it suggests the evidence-base for this significant medical education activity has not been robustly critiqued. Given the frequency of new medical schools being established around the world along with the high political and financial stakes and complexity of the task, the potential for inefficient or ineffective practices without a strong evidence-base is of concern, and a compelling argument for a comprehensive review despite the broad scope can be made.

We sought to address this information gap through a scoping review of the literature. Our scoping review asked two research questions: (1) *What is the nature of the available literature on establishing a new medical school?*; (2) *What are the key factors to be considered when establishing a new medical school?* By answering these questions, we aimed to map the nature of current evidence available to assist future founding leaders, explicate key elements for consideration during establishment, and fill a publication gap.

## Methods

We chose to conduct a scoping review since they are useful for identifying and synthesising the key aspects of a broad concept and have great utility in mapping the size, variety, and nature of the existing literature, particularly when a topic has not been reviewed before or when the information is likely to be broad, complex, and heterogenous (Arksey & O'Malley, 2005; Munn et al., 2018; Peters et al., 2015, 2020; Thomas et al., 2017; Tricco et al., 2018). We followed defined scoping review methodology: (1) identify the review research questions (presented above); (2) identify relevant studies/articles; (3) select the studies/articles; (4) chart the data; (5) collate, summarise, and report results (Arksey & O'Malley, 2005; Levac et al., 2010; Peters et al., 2020; Thomas et al., 2017).

### Identifying relevant studies/articles

We searched multiple databases at two time points—May 2015 and January 2021—including Ovid MEDLINE(R) In-Process & Other Non-Indexed Citations, Ovid MEDLINE(R) 1946 to Present, Scopus, Web of Science, ProQuest, and the Cochrane Library of Systematic Reviews. The same search string was used for each database, applying appropriate syntax for adjacency operators:

$$\begin{gathered} \left( {medic*} \right) \, adj2 \, \left( {school* \, OR \, college* \, OR \, program* \, OR \, course*} \right) \, adj2 \, (new \, OR \, inaugural* \, OR \hfill \\ first \, OR \, initial*) \hfill \\ {\text{AND}} \hfill \\ (establish* \, OR \, set* \, up \, OR \, found* \, OR \, creat* \, OR \, plan* \, OR \, commenc* \, OR \, build* \, OR \, design* \hfill \\ OR \, start*) \hfill \\ \end{gathered}$$

Searches were limited to English-language articles only, but no limits were set on publication types and grey literature was included. No limits were set on publication dates for the 2015 search, but publication dates were limited to ‘2015 to current’ in 2021. Further articles were identified through manual processes such as bibliographic searches, online searches (such as Google and Google Scholar searches), journal content alerts, medical school websites, and personal contacts. Search results were collated into EndNote X9 software.

### Selecting the studies/articles

Inclusion or exclusion of articles was based on their suitability in answering the review questions rather than on clear-cut methodological criteria or critical appraisal checklists (Eva, 2009; Harden et al., 1999; Pawson et al., 2005; Yardley & Dornan, 2012). This is a common approach for reviews of complex educational interventions that are not easily amenable to methodological nor contextual standardisation (Education Group for Guidelines on Evaluation, 1999; Eva, 2009; Harden et al., 1999; Pawson et al., 2005; Yardley & Dornan, 2012). It is also consistent with scoping review methodology where inclusion and exclusion criteria can be flexibly devised both a priori as well as post hoc, taking advantage of increasing familiarity with the literature to determine relevance (Arksey & O'Malley, 2005; Levac et al., 2010; Sucharew & Macaluso, 2019; Thomas et al., 2017) and “best fit” for the review questions (Arksey & O'Malley, 2005, p. 26). Iteratively parsing through titles, abstracts, and full-texts multiple times developed this familiarity to determine relevance. Uncertainties were also iteratively addressed as our inclusion and exclusion criteria were increasingly refined.

**Fig. 1:**
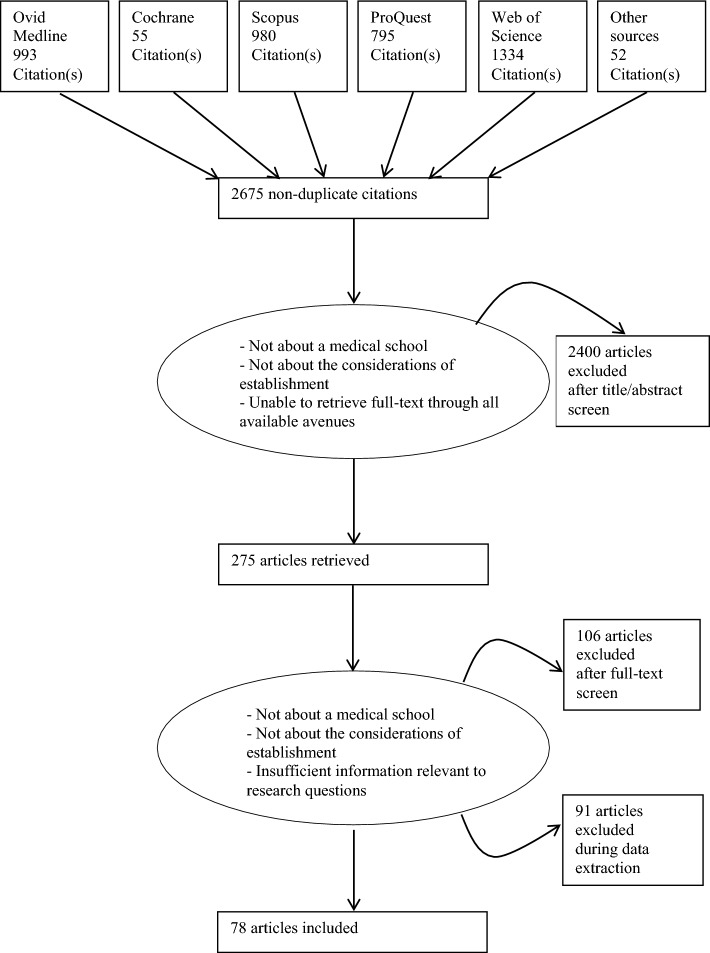
PRISMA flow chart

Articles were included if they pertained to the overarching considerations of establishing a new medical school; or contributed vital information for the research questions even if only focused on a single aspect of a new medical school’s functioning. Articles were excluded if they were not about a new medical school; not about the factors and processes involved with the establishment of one; or we were unable to retrieve the full-text articles through all the available online and physical search methods and library services. Using these criteria, initially 118 articles were included in this review, including forty (40) articles published prior to the year 2000. As analysis proceeded, it became evident that the articles published last century did not provide any additional insight. Thus, to gain the most relevant and contemporary perspectives of new medical school establishment, only articles published in the new millennium (i.e., between 2000 and 2021) were finally included (n = 78) (see Fig. 1).

### Charting the data

Charting the data involved examining the retrieved articles for the following extraction fields: authors; year of publication; aim/purpose of article; type of article (e.g., advice/opinion, report, accreditation standards, research, etc.); research methodology used; theoretical framework identified; and context/country being written about. The data was compiled into a Microsoft Excel spreadsheet (see Supplemental Digital Appendix).

### Collating, summarising, and reporting the results

Following a two-part approach described by Arksey and O’Malley (2005, pp. 27–28), collating and summarising the data were split into two parts to answer each of the two review research questions. The first part sought to understand the nature and variety of the literature by using Microsoft Excel to sort, count, and structurally analyse all the articles (n = 78) (see Supplemental Digital Appendix).

The second part comprised a thematic analysis of the literature to produce a ‘narrative’ review of the topic (Arksey & O'Malley, 2005; Eva, 2008; Kiger & Varpio, 2020). We followed an iterative process through combined deductive and inductive initial open-ended, provisional coding (first-cycle coding) followed by rearranging and reclassifying codes (second-cycle coding) (Saldaña, 2009) using software such as EndNote X9, Microsoft Word, Microsoft Excel, and XMind Pro 7. Initial broad deductive categories such as ‘costs’, ‘staff’, ‘curriculum’, ‘students’, ‘buildings’, and ‘accreditation’ were identified through brainstorming the economic, human, material, and educational factors to be considered when establishing a new medical school and conceptually mapped using XMindPro 7. Articles were read multiple times and key sections were annotated in EndNote X9. Extensive tables combining codes were categorised using Microsoft Word and Microsoft Excel. Codes were iteratively refined and compared with findings in the articles, This process enabled themes to be identified that we classified as thirteen key considerations when establishing new medical schools and also revealed further gaps in the literature.

## Results

In answer to our first research question regarding the nature of the available literature on new medical school establishment, we particularly examined the ‘type of article’ extraction field (see Supplemental Digital Appendix, ‘Type’ column). The tallies in Table 1 show that most articles that have been published on the topic are descriptive in nature. Most articles were written by founding deans or other leaders of new medical schools, outlining personal and institutional experiences without report of research methodologies nor underpinning theoretical frameworks.

**Table 1:** Types of publications on new medical school establishment

Type	Number
Advice/opinions from experienced experts	7 (9%)
Reports from specific new medical schools	37 (47%)
Reports from an authoritative organisation	4 (5%)
Discussion of national/regional situation	6 (8%)
Discussion of global situation	3 (4%)
Accreditation standards & guidelines	6 (8%)
Research articles	10 (13%)
Other (letter-to-editor, news article, prospectus, proposal, historical account, commentary, etc.)	5 (6%)
Total	78 (100%)

Advice/opinion articles outlined the perspectives of leaders who had been involved with establishing one or more new medical schools (n = 7, 9%). Reports from specific new medical schools described one or several aspects of establishment such as staffing, curriculum design, or admissions procedures (n = 37, 47%). Reports from authoritative organisations came from entities such as the World Federation for Medical Education (reporting their efforts to define international standards) (World Federation for Medical Education, 2000); the Association of American Medical Colleges (summarising the experiences of sixteen new medical schools) (Association of American Medical Colleges, 2012); the Texas Higher Education Coordinating Board (briefly overviewing steps to establishing a new medical school) (Texas Higher Education Coordinating Board, 2008); and the Australian Medical Council (who collated the key accreditation challenges facing new medical schools) (Field, 2011) (n = 4, 5%). Discussions of the national/regional situation summarised issues such as workforce shortages and maldistribution; medical education trends; medical school or graduate numbers; and prospects for new medical schools (Mullan, 2003; Pericleous, 2011; Reis et al., 2009; Sabde et al., 2020; Salter et al., 2016; Smith, 2009) (n = 6, 8%). Similar issues were discussed in the papers on the global situation (Frenk et al., 2010; Karle, 2010; Rizwan et al., 2018) (n = 3, 4%). Accreditation guidelines listed standards that were categorised into various domains such as mission and values; educational program; student assessment; admissions processes; staffing; evaluation; and governance and administration (Australian Medical Council, 2012; Liaison Committee on Medical Education, 2006, 2008, 2020; World Federation for Medical Education, 2000, 2015, 2020) (n = 6, 8%).

Several of the research articles (n = 10; 13%) related primarily to a single aspect of a new medical school such as staff retention factors (Nausheen et al., 2018), student experiences (Delgado et al., 2017), or a specific curriculum element (Colquhoun et al., 2009; Lockyer & Patterson, 2005). A large multiple case study commissioned by the Josiah Macy, Jr. Foundation on twenty-nine new medical schools established in the United States produced several articles and reports (Whitcomb, 2009, 2010, 2013, 2018). Two sources were reported as retrospective single case studies (Cristobal & Worley, 2012; Tesson et al., 2009), but did not describe formal case study research methods (Yin, 2014). It could also be argued that reports from specific new medical schools were a type of single case study even if not explicitly noted as such. None of the new medical schools reported a research methodology associated with their process of establishment. Furthermore, none of the research articles proposed a theoretical framework that could underpin the overarching process.

**Fig. 2:**
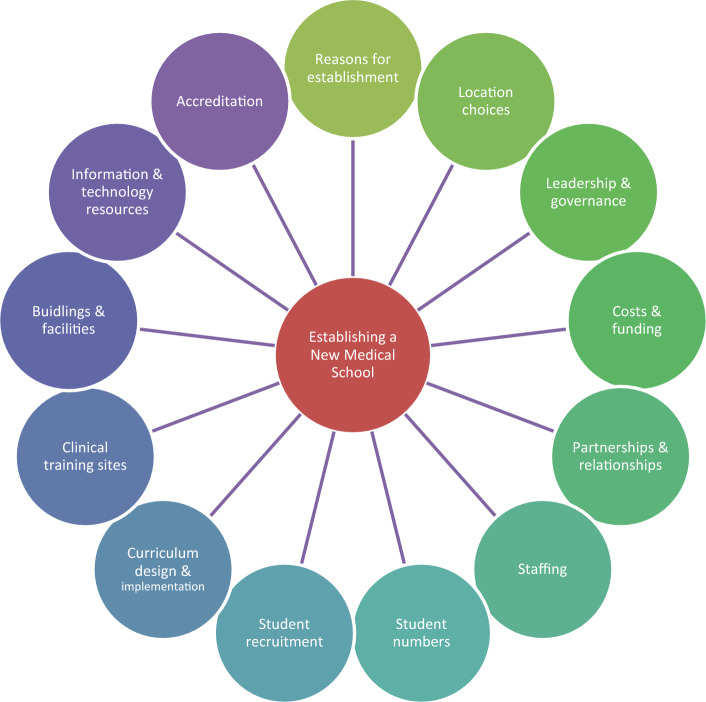
Thirteen key considerations of new medical school establishment (13KCs)

In answer to our second research question regarding the factors involved with establishing a new medical school, we identified thirteen key considerations. These included: reasons for establishment; location choices; leadership and governance; costs and funding; partnerships; staffing; student numbers; student recruitment; curriculum design and implementation; clinical training sites; buildings and facilities; information and technology resources; and accreditation. There was no specific order nor hierarchy to these considerations as highlighted in Fig. 2. We discuss each of the considerations in turn.

### Reasons for establishment

Almost ubiquitously, addressing doctor workforce shortages and maldistributions were cited as reasons for establishing a new medical school (Association of American Medical Colleges, 2012; Bin Abdulrahman & Saleh, 2015; Castelo-Branco et al., 2016; Cathcart-Rake et al., 2017; Condon et al., 2017; Drobac & Morse, 2016; Fogarty et al., 2012; Furukawa et al., 2017; Hays & Sen Gupta, 2003; Hays et al., 2003; Howe et al., 2004; Hurt & Harris, 2005; Lanphear & Strasser, 2008; Lawrenson et al., 2017; Lawson et al., 2004; Lockyer & Patterson, 2005; Mokone et al., 2014; Olds & Barton, 2015; Penner, 2018; Pinder et al., 2008; Reis et al., 2009; Rizwan et al., 2018; Salter et al., 2016; Smego et al., 2010; Smith, 2009; Strasser & Lanphear, 2008; Strasser et al., 2009; Tesson et al., 2009; University of California Riverside, 2008; Whitcomb, 2009; Worley et al., 2019). Other reasons included improving health services/systems; responding to community health needs; developing the community economically, intellectually, educationally, and socio-culturally; enacting social missions of inclusion, diversity, minority disadvantage, and ethnic disparities; pursuing research mandates; implementing medical education innovation and reform; improving university or health facility reputation; pursuing profit, philanthropic, or religious goals; and attracting local diaspora back or other well-qualified personnel to the region (Association of American Medical Colleges, 2012; Bin Abdulrahman & Saleh, 2015; Cookson, 2013; Cristobal & Worley, 2012; Drobac & Morse, 2016; Eichbaum et al., 2014a, b; Fogarty et al., 2012; Frenk et al., 2010; Hamdy & Anderson, 2006; Härtl et al., 2017; Hays, 2001, 2018; Hays et al., 2003, 2019a, b; Howe et al., 2004; Hurt & Harris, 2005; Karle, 2010; Lanphear & Strasser, 2008; Lawrenson et al., 2017; Lawson et al., 2004; Lockyer & Patterson, 2005; Mullan, 2003; Muula, 2006; Nausheen et al., 2018; Olds & Barton, 2015; Penner, 2018; Pericleous, 2011; Reis et al., 2009; Romano, 2001; Sabde et al., 2020; Salter et al., 2016; Schuster et al., 2020; Simoyan et al., 2011; Smego et al., 2010; Smith, 2009; Snadden et al., 2011; Strasser & Lanphear, 2008; Strasser et al., 2009; Tesson et al., 2009; University of California Riverside, 2008; Whitcomb, 2009, 2010, 2013, 2018, 2020; Williams et al., 2008; World Federation for Medical Education, 2015; Worley et al., 2019). Reasons to NOT proceed with establishment included cost; expediency; small population, scarce resources, inability to procure sufficient funds, clinical affiliations, and/or preliminary accreditation; and the fear that it might cause a future glut of doctors (Condon et al., 2017; Frenk et al., 2010; Furukawa et al., 2017; Karle, 2010; Lawrenson et al., 2017; McFee & Aust, 2005; Mokone et al., 2014; Muula, 2006; Norris et al., 2006; Pericleous, 2011; Romano, 2001; Salter et al., 2016; Texas Higher Education Coordinating Board, 2008; Whitcomb, 2009, 2010, 2013, 2018).

### Location choices

Location choices were influenced by reasons for establishment and availability of resources like clinical training sites and staff. Thus, new medical schools were commonly located in areas of workforce shortage; within the communities they were intended to serve; distributed across multiple campuses/cities/regions utilising various health service facilities for clinical training; co-located with the parent university but also sometimes in satellite countries/continents different from the parent university; or in a location central to multi-organisational or multi-regional collaborations (Association of American Medical Colleges, 2012; Bin Abdulrahman & Saleh, 2015; Cathcart-Rake et al., 2017; Chavez et al., 2012; Colquhoun et al., 2009; Condon et al., 2017; Cookson, 2013; Cristobal & Worley, 2012; Delgado et al., 2017; Drobac & Morse, 2016; Field, 2011; Fogarty et al., 2012; Frenk et al., 2010; Furukawa et al., 2017; Gifford, 2007; Hamdy & Anderson, 2006; Härtl et al., 2017; Hays, 2001; Hays & Sen Gupta, 2003; Hays et al., 2003, 2019a, b; Howe et al., 2004; Hurt & Harris, 2005; Karle, 2010; Kebaetse et al., 2014; Lanphear & Strasser, 2008; Lawrenson et al., 2017; Lawson et al., 2004; Lockyer & Patterson, 2005; Mangan, 2009; McFee & Aust, 2005; Mokone et al., 2014; Nonaillada, 2020; Norris et al., 2006; Olds & Barton, 2015; Penner, 2018; Reis et al., 2009; Salter et al., 2016; Schuster et al., 2020; Simoyan et al., 2011; Smego et al., 2010; Smith, 2009; Snadden et al., 2011; Strasser & Lanphear, 2008; Strasser et al., 2009; Tesson et al., 2009; University of California Riverside, 2008; Whitcomb, 2009, 2013, 2018, 2020; Williams et al., 2008; Worley et al., 2019).

### Leadership and governance

Crucial early steps were to hire the founding dean (or equivalent); appoint the leadership team; and set up steering committees and sub-committees (Association of American Medical Colleges, 2012; Bin Abdulrahman & Saleh, 2015; Cookson, 2013; Hamdy & Anderson, 2006; Härtl et al., 2017; Hays et al., 2019a, b; Hurt & Harris, 2005; Khalil & Kibble, 2014; Lanphear & Strasser, 2008; Liaison Committee on Medical Education, 2006, 2008, 2020; Olds & Barton, 2015; Penner, 2018; Schuster et al., 2020; Strasser & Lanphear, 2008; Strasser et al., 2009; Tesson et al., 2009; University of California Riverside, 2008; Whitcomb, 2009, 2013, 2018; World Federation for Medical Education, 2015, 2020; Worley et al., 2019). The Founding Dean was a lynchpin, responsible for leading the whole team, coordinating all processes, and addressing all the considerations (Bin Abdulrahman & Saleh, 2015; Cookson, 2013; Hamdy & Anderson, 2006; Liaison Committee on Medical Education, 2006; University of California Riverside, 2008; Whitcomb, 2009, 2018). Other key appointments included leaders to oversee aspects such as administration; finances; fund raising; curriculum; staffing; clinical affiliations; student admissions; student support; research; information technology; and business planning (Bin Abdulrahman & Saleh, 2015; Liaison Committee on Medical Education, 2006, 2008; Mokone et al., 2014).

Members of the founding team needed characteristics such as belief in the mission; shared vision; strategic flexibility; tenacity; courage; enthusiasm; willingness to work with all partners; ability to overcome challenges; and energy to work tirelessly (Association of American Medical Colleges, 2012; Castelo-Branco et al., 2016; Reis et al., 2009; Snadden et al., 2011). They needed diverse skills and experience in leadership; communication; teamwork; governance and administration; resource management; stakeholder engagement; faculty development; research; and medical education (Association of American Medical Colleges, 2012; Bin Abdulrahman & Saleh, 2015; Cookson, 2013; Härtl et al., 2017; Hays et al., 2019a, b; Khalil & Kibble, 2014; Lawson et al., 2004; Liaison Committee on Medical Education, 2008, 2020; Reis et al., 2009; University of California Riverside, 2008). They also needed support and training in leadership; team process; curriculum design; educational methodologies; research; and equity, inclusion, and diversity (Khalil & Kibble, 2014; Liaison Committee on Medical Education, 2008, 2020; Nonaillada, 2020; Schuster et al., 2020).

### Costs and funding

Medical education is very expensive with a world average estimated expenditure of US$122,000 per medical graduate, ranging from US$14,000 (in China) to US$497,000 (in North America) (Drobac & Morse, 2016; Frenk et al., 2010; Hays, 2001; Hays et al., 2019a, b; Hurt & Harris, 2005; Mokone et al., 2014; Mullan, 2003; Muula, 2006; Norris et al., 2006; Pericleous, 2011; Romano, 2001; Strasser et al., 2009; Texas Higher Education Coordinating Board, 2008; Whitcomb, 2009, 2010). For a new medical school, start-up or establishment costs ranged between US$75 million and US$150 million; and running or maintenance costs ranged between US$12 million and US$168 million (Bin Abdulrahman & Saleh, 2015; Frenk et al., 2010; Hays, 2001; Hurt & Harris, 2005; Norris et al., 2006; Pericleous, 2011; Tesson et al., 2009; Texas Higher Education Coordinating Board, 2008; University of California Riverside, 2008; Whitcomb, 2009, 2013). Rising costs of high quality modern medical education have contributed to global inequities and inequalities and lower cost solutions are required (Cathcart-Rake et al., 2017; Chavez et al., 2012; Cookson, 2013; Cristobal & Worley, 2012; Drobac & Morse, 2016; Frenk et al., 2010; Hays et al., 2019a, b; Karle, 2010; Rizwan et al., 2018; Simoyan et al., 2011).

Finding adequate funds was a significant challenge for many founding teams (Eichbaum et al., 2014a; Frenk et al., 2010; Karle, 2010; Whitcomb, 2009, 2010, 2013, 2018, 2020). Multiple sources—both public and private—were usually required (Association of American Medical Colleges, 2012; Frenk et al., 2010; Hays et al., 2019a, b; Karle, 2010; Liaison Committee on Medical Education, 2008, 2020; University of California Riverside, 2008; Whitcomb, 2009, 2013) and included federal and state government support; university funding; development grants; research funding; philanthropic donations; endowments and bequests; international aid; student tuition fees; clinical revenue subsidies; and community fund-raising (Association of American Medical Colleges, 2012; Cristobal & Worley, 2012; Eichbaum et al., 2014a; Eichbaum et al., 2015; Fogarty et al., 2012; Frenk et al., 2010; Hamdy & Anderson, 2006; Hays et al., 2003; Hays et al., 2019a, b; Hurt & Harris, 2005; Lanphear & Strasser, 2008; Liaison Committee on Medical Education, 2008, 2020; Mokone et al., 2014; Norris et al., 2006; Pericleous, 2011; Simoyan et al., 2011; Smego et al., 2010; Snadden et al., 2011; Strasser & Lanphear, 2008; Strasser et al., 2009; Tesson et al., 2009; University of California Riverside, 2008; Whitcomb, 2009, 2013, 2018; Williams et al., 2008; Worley et al., 2019). Partnerships between stakeholders could help procure sufficient funds (Association of American Medical Colleges, 2012; Howe et al., 2004; Tesson et al., 2009; Whitcomb, 2009, 2013, 2018) and sharing in-kind resources such as clinical, academic, research personnel, or physical spaces could mitigate financial outlays (Cathcart-Rake et al., 2017; Hamdy & Anderson, 2006; Hays et al., 2019a, b; Lawson et al., 2004; Liaison Committee on Medical Education, 2020; Tesson et al., 2009; Whitcomb, 2020).

### Partnerships

Cooperation and collaboration of many internal and external stakeholders were needed, including university councils, clinical training sites, health service entities, governmental authorities, regulatory bodies, funding sources, specialty training bodies, professional associations, other health professions, students, staff, Indigenous peoples, other minority groups, health consumers, and members of the public (Association of American Medical Colleges, 2012; Australian Medical Council, 2012; Frenk et al., 2010; Hays, 2001; Hays et al., 2019a, b; Lanphear & Strasser, 2008; Smego et al., 2010; Strasser & Lanphear, 2008; Strasser et al., 2009; University of California Riverside, 2008; Whitcomb, 2009, 2013, 2018; World Federation for Medical Education, 2015, 2020). Effectively engaging the varied stakeholders was quite challenging (Association of American Medical Colleges, 2012; Frenk et al., 2010; Hays et al., 2019a, b; Penner, 2018; Snadden et al., 2011; Whitcomb, 2013) and required finding common ground, developing shared vision, mission, and goals; creating win–win relationships; promoting ownership; well-articulated guiding principles; good communication and reporting; in-person relationship building; continuous nurturing; and formal agreements incorporating conflict resolution mechanisms (Association of American Medical Colleges, 2012; Australian Medical Council, 2012; Cookson, 2013; Field, 2011; Kebaetse et al., 2014; Lanphear & Strasser, 2008; Liaison Committee on Medical Education, 2006, 2008, 2020; Penner, 2018; Snadden et al., 2011; Whitcomb, 2013; World Federation for Medical Education, 2000, 2015, 2020).

### Staffing

Staff were an “ultimate resource” (Frenk et al., 2010, p. 1941) and included academic, clinical, research, and administrative personnel (Association of American Medical Colleges, 2012; Australian Medical Council, 2012; Cookson, 2013; Drobac & Morse, 2016; Karle, 2010; Liaison Committee on Medical Education, 2006, 2008, 2020; Texas Higher Education Coordinating Board, 2008; University of California Riverside, 2008; Whitcomb, 2009; World Federation for Medical Education, 2015, 2020). Recruiting strategies included advertising in local, regional, national, and international newspapers, medical journals, and academic websites; local networking; part-time or joint appointments, allowing clinicians to also continue in clinical practice; sharing staff amongst partner medical schools without compromising either; and appointing volunteer adjunct or affiliate faculty (Association of American Medical Colleges, 2012; Cookson, 2013; Cristobal & Worley, 2012; Eichbaum et al., 2015, 2014a, b; Field, 2011; Fogarty et al., 2012; Hays et al., 2019a, b; Liaison Committee on Medical Education, 2008; McDonald et al., 2014; McFee & Aust, 2005; Mokone et al., 2014; Norris et al., 2006; Olds & Barton, 2015; Sabde et al., 2020; Simoyan et al., 2011; Smego et al., 2010; Snadden et al., 2011; Whitcomb, 2009; Williams et al., 2008). Recruiting and retaining staff in sufficient numbers; of the right calibre; and with the desired representation of diversity was a significant challenge for many new medical schools (Association of American Medical Colleges, 2012; Bonner et al., 2018; Cookson, 2013; Drobac & Morse, 2016; Eichbaum et al., 2014a; Field, 2011; Frenk et al., 2010; Howe et al., 2004; Hurt & Harris, 2005; Karle, 2010; McDonald et al., 2014; Mokone et al., 2014; Nausheen et al., 2018; Norris et al., 2006; Reis et al., 2009; Smego et al., 2010; Smith, 2009; Snadden et al., 2011; Tesson et al., 2009; University of California Riverside, 2008; Whitcomb, 2009). Given the pioneering nature of new medical schools, recruiting resilient, adaptable, and dedicated faculty was paramount (Association of American Medical Colleges, 2012; Cookson, 2013; Hamdy & Anderson, 2006).

### Student numbers

Class size decisions depended on factors in the local context such as population size; health system status; workforce needs; the pool of eligible applicants; numbers of local students studying medicine elsewhere; cost-effectiveness; and the adequacy of available resources including funding, facilities, and staff (Australian Medical Council, 2012; Bin Abdulrahman & Saleh, 2015; Cathcart-Rake et al., 2017; Hays et al., 2019a, b; Hurt & Harris, 2005; Karle, 2010; Liaison Committee on Medical Education, 2008; Olds & Barton, 2015; Pericleous, 2011; Reis et al., 2009; Snadden et al., 2011; Whitcomb, 2009; World Federation for Medical Education, 2015, 2020). Suggestions included “200–300 graduates per year within an acceptable range of 50–500” (2010, p. 166) or “an initial cohort of between 60 and 100” (2019, p. 399). Individual institutions reported between 25 and 152 students in their charter classes (Association of American Medical Colleges, 2012; Cathcart-Rake et al., 2017; Cristobal & Worley, 2012; Delgado et al., 2017; Fogarty et al., 2012; Hamdy & Anderson, 2006; Hays, 2001; Howe et al., 2004; Hurt & Harris, 2005; Mangan, 2002, 2009; Mokone et al., 2014; Nausheen et al., 2018; Pericleous, 2011; Schuster et al., 2020; Simoyan et al., 2011; Smego et al., 2010; Strasser & Lanphear, 2008; Strasser et al., 2009; Tesson et al., 2009; University of California Riverside, 2008; Whitcomb, 2009, 2010, 2013, 2018), while new branch campuses of existing medical schools reported between 8 and 32 students in their initial cohorts (Cathcart-Rake et al., 2017; Pinder et al., 2008; Williams et al., 2008; Worley et al., 2019). Many proposed to quickly scale their class sizes up in subsequent years (Association of American Medical Colleges, 2012; Fogarty et al., 2012; Hays, 2001; Howe et al., 2004; Hurt & Harris, 2005; Mangan, 2002; Mokone et al., 2014; Nausheen et al., 2018; Smego et al., 2010; University of California Riverside, 2008; Whitcomb, 2009), and some increased by fifty percent in consecutive years (Association of American Medical Colleges, 2012; Hurt & Harris, 2005; Nausheen et al., 2018; Texas Higher Education Coordinating Board, 2008; Whitcomb, 2009, 2013; Williams et al., 2008).

### Student recruitment

Admissions policies and procedures needed to reflect institutional missions and purposes and needed to be transparent, clear, and evidence-based (Association of American Medical Colleges, 2012; Australian Medical Council, 2012; Bin Abdulrahman & Saleh, 2015; Eichbaum et al., 2014a, b; Frenk et al., 2010; Hurt & Harris, 2005; Karle, 2010; Liaison Committee on Medical Education, 2006, 2008, 2020; Schuster et al., 2020; Snadden et al., 2011; Strasser et al., 2009; Tesson et al., 2009; World Federation for Medical Education, 2000, 2015, 2020). Admissions criteria usually included a varied combination of aptitude scores; national entrance examination scores; academic achievement scores such as high school or university Grade Point Averages; performance in preparatory courses; interview performance; and personal statements (Bin Abdulrahman & Saleh, 2015; Cathcart-Rake et al., 2017; Cristobal & Worley, 2012; Howe et al., 2004; Lawson et al., 2004; Sabde et al., 2020; Schuster et al., 2020; Strasser & Lanphear, 2008; Strasser et al., 2009; Tesson et al., 2009; University of California Riverside, 2008; World Federation for Medical Education, 2015, 2020). Inherent inequalities and bias in certain admissions methodologies that privileged urban and affluent applicants—such as academic merit, standardised testing, and privatisation—needed to be acknowledged and accounted for (Cathcart-Rake et al., 2017; Condon et al., 2017; Eichbaum et al., 2015, 2014a, b; Frenk et al., 2010; Karle, 2010; Olds & Barton, 2015; Strasser & Lanphear, 2008; Strasser et al., 2009; Tesson et al., 2009; World Federation for Medical Education, 2015, 2020). Many medical schools and accreditation standards emphasised the need to make explicit entry provisions for equity, diversity, rurality, minorities, under-served populations, and local applicants, however, discrimination and bias were to be guarded against other than for the purposes of deliberate affirmative action (Association of American Medical Colleges, 2012; Australian Medical Council, 2012; Cristobal & Worley, 2012; Drobac & Morse, 2016; Eichbaum et al., 2015, 2014a, b; Fogarty et al., 2012; Frenk et al., 2010; Hays et al., 2003; Howe et al., 2004; Hurt & Harris, 2005; Karle, 2010; Lanphear & Strasser, 2008; Lawson et al., 2004; Liaison Committee on Medical Education, 2006, 2008, 2020; Nausheen et al., 2018; Olds & Barton, 2015; Salter et al., 2016; Schuster et al., 2020; Simoyan et al., 2011; Snadden et al., 2011; Strasser & Lanphear, 2008; Strasser et al., 2009; Tesson et al., 2009; University of California Riverside, 2008; World Federation for Medical Education, 2000, 2015, 2020).

### Curriculum design and implementation

Curriculum decisions needed to align with the vision/mission/objective of the new medical school and with the available educational resources and clinical services (Colquhoun et al., 2009; Cookson, 2013; Cristobal & Worley, 2012; Hamdy & Anderson, 2006; Hays, 2001; Hays & Sen Gupta, 2003; Hays et al., 2003, 2019a, b; Howe et al., 2004; Hurt & Harris, 2005; Kebaetse et al., 2014; Liaison Committee on Medical Education, 2006, 2008, 2020; Snadden et al., 2011; Strasser & Lanphear, 2008; Tesson et al., 2009; World Federation for Medical Education, 2000, 2015, 2020). Curriculum discussions emphasised ‘outcomes’ and ‘competencies’ such as patient-centred care; communication skills; knowledge application; technical skills; clinical reasoning; evidence-based practice; quality improvement; interdisciplinary teamwork; public health promotion; research skills; critical inquiry; life-long learning; management and leadership capabilities; reflective practise; and socially responsible professionalism (Bin Abdulrahman & Saleh, 2015; Castelo-Branco et al., 2016; Cristobal & Worley, 2012; Eichbaum et al., 2015, 2014a, b; Frenk et al., 2010; Hamdy & Anderson, 2006; Härtl et al., 2017; Khalil & Kibble, 2014; Lockyer & Patterson, 2005; Smith, 2009).

Many new medical schools used the opportunity to innovatively construct their curriculum themselves (Association of American Medical Colleges, 2012; Smith, 2009; Strasser & Lanphear, 2008; Strasser et al., 2009; Tesson et al., 2009; Whitcomb, 2009), while others accessed pre-existing medical curricula and modified it for their contexts (Castelo-Branco et al., 2016; Lawson et al., 2004; Mokone et al., 2014; Pericleous, 2011; Snadden et al., 2011). There was a trend away from ‘traditional’ models of curriculum to ‘integrated’ models (Bin Abdulrahman & Saleh, 2015; Castelo-Branco et al., 2016; Eichbaum et al., 2015; Frenk et al., 2010; Hamdy & Anderson, 2006; Howe et al., 2004; Khalil & Kibble, 2014; Lawrenson et al., 2017). There was also an increasing trend towards community-based ‘longitudinal integrated clerkships’ and curricula emphasising primary care (Association of American Medical Colleges, 2012; Condon et al., 2017; Cristobal & Worley, 2012; Hays, 2001; Hays et al., 2003, 2019a, b; Hurt & Harris, 2005; Lawson et al., 2004; Smego et al., 2010; Strasser & Lanphear, 2008; Strasser et al., 2009; Tesson et al., 2009; University of California Riverside, 2008). Adherence to out-dated and overloaded curricula were major challenges for new medical schools (Frenk et al., 2010; Karle, 2010; World Federation for Medical Education, 2000, 2015, 2020).

### Clinical training sites

Good clinical encounters were essential with direct patient care of adequate numbers of ambulatory and hospitalised patients; a broad case mix of health and illness presentations; in a range of primary care, tertiary hospital, and community settings (Association of American Medical Colleges, 2012; Australian Medical Council, 2012; Cathcart-Rake et al., 2017; Colquhoun et al., 2009; Cristobal & Worley, 2012; Drobac & Morse, 2016; Eichbaum et al., 2015, 2014a, b; Field, 2011; Fogarty et al., 2012; Frenk et al., 2010; Hamdy & Anderson, 2006; Hays, 2001; Hays et al., 2003, 2019a, b; Howe et al., 2004; Hurt & Harris, 2005; Karle, 2010; Lanphear & Strasser, 2008; Lawrenson et al., 2017; Lawson et al., 2004; Liaison Committee on Medical Education, 2006, 2008, 2020; Mangan, 2009; Mokone et al., 2014; Norris et al., 2006; Salter et al., 2016; Schuster et al., 2020; Smego et al., 2010; Snadden et al., 2011; Strasser & Lanphear, 2008; Strasser et al., 2009; Tesson et al., 2009; University of California Riverside, 2008; Whitcomb, 2009, 2013, 2018; World Federation for Medical Education, 2000, 2015, 2020; Worley et al., 2019). Formal affiliations with a wide range of public and private health services had to be developed; clinical training sites had to be accredited as teaching locations; and health service staff needed support and training for their educational roles (Association of American Medical Colleges, 2012; Australian Medical Council, 2012; Castelo-Branco et al., 2016; Cathcart-Rake et al., 2017; Colquhoun et al., 2009; Condon et al., 2017; Cookson, 2013; Cristobal & Worley, 2012; Fogarty et al., 2012; Frenk et al., 2010; Härtl et al., 2017; Hays et al., 2019a, b; Hurt & Harris, 2005; Karle, 2010; Khalil & Kibble, 2014; Lanphear & Strasser, 2008; Lawson et al., 2004; Liaison Committee on Medical Education, 2006, 2008, 2020; Mokone et al., 2014; Nonaillada, 2020; Norris et al., 2006; Olds & Barton, 2015; Smego et al., 2010; Snadden et al., 2011; Strasser & Lanphear, 2008; Tesson et al., 2009; University of California Riverside, 2008; Whitcomb, 2009, 2010, 2013, 2018, 2020; Williams et al., 2008; World Federation for Medical Education, 2000, 2015, 2020).

Historically, tertiary teaching hospitals were the primary sites for clinical placements, however, medical schools were increasingly utilising more community-based health facilities with research evidence that smaller student groups at a site were better than larger groups, and that smaller rural sites can provide effective clinical training (Association of American Medical Colleges, 2012; Bin Abdulrahman & Saleh, 2015; Colquhoun et al., 2009; Condon et al., 2017; Cookson, 2013; Drobac & Morse, 2016; Fogarty et al., 2012; Frenk et al., 2010; Hamdy & Anderson, 2006; Härtl et al., 2017; Hays et al., 2003, 2019a, b; Howe et al., 2004; Hurt & Harris, 2005; Karle, 2010; Kebaetse et al., 2014; Lanphear & Strasser, 2008; Lawrenson et al., 2017; Lawson et al., 2004; Lockyer & Patterson, 2005; Mangan, 2009; McFee & Aust, 2005; Mokone et al., 2014; Norris et al., 2006; Olds & Barton, 2015; Strasser & Lanphear, 2008; Strasser et al., 2009; Tesson et al., 2009; University of California Riverside, 2008; Whitcomb, 2009, 2013, 2018). Clinical training could also be supported by clinical skills laboratories, simulated patients, and mannequin simulations (Association of American Medical Colleges, 2012; Bin Abdulrahman & Saleh, 2015; Cathcart-Rake et al., 2017; Frenk et al., 2010; Hurt & Harris, 2005; Lawson et al., 2004; Liaison Committee on Medical Education, 2008; Smego et al., 2010; Snadden et al., 2011; Strasser et al., 2009; Tesson et al., 2009; University of California Riverside, 2008; Whitcomb, 2009, 2013; World Federation for Medical Education, 2000, 2015, 2020). Ensuring adequate quantity, and quality of clinical encounters, clinical teachers, and clinical facilities were significant challenges and a number of institutions that considered establishing a new medical school could not proceed due to difficulty securing these (Association of American Medical Colleges, 2012; Colquhoun et al., 2009; Eichbaum et al., 2014a; Field, 2011; Karle, 2010; Lanphear & Strasser, 2008; Nausheen et al., 2018; Norris et al., 2006; Whitcomb, 2013, 2018; Williams et al., 2008).

### Buildings and facilities

Providing adequate physical facilities included administrative, instructive, research, and social spaces; educational, clinical, technological, and research equipment; and could range from quite rudimentary to highly sophisticated (Association of American Medical Colleges, 2012; Australian Medical Council, 2012; Bin Abdulrahman & Saleh, 2015; Cookson, 2013; Field, 2011; Fogarty et al., 2012; Frenk et al., 2010; Hurt & Harris, 2005; Kebaetse et al., 2014; Liaison Committee on Medical Education, 2006, 2008, 2020; Norris et al., 2006; Smego et al., 2010; Snadden et al., 2011; Strasser et al., 2009; Tesson et al., 2009; University of California Riverside, 2008; Whitcomb, 2009, 2013, 2018; Williams et al., 2008; World Federation for Medical Education, 2000, 2015, 2020; Worley et al., 2019). Instructional spaces included small group tutorial rooms; large group lecture theatres; independent study spaces; clinical skills laboratories including simulation and mock consultation facilities; multi-purpose laboratories for anatomy, physiology, biochemistry, histology, and pathology; libraries; and computer rooms (Association of American Medical Colleges, 2012; Bin Abdulrahman & Saleh, 2015; Cookson, 2013; Fogarty et al., 2012; Hurt & Harris, 2005; Kebaetse et al., 2014; Liaison Committee on Medical Education, 2006, 2008; Norris et al., 2006; Smego et al., 2010; Snadden et al., 2011; Strasser et al., 2009; Tesson et al., 2009; University of California Riverside, 2008; Whitcomb, 2009, 2013; World Federation for Medical Education, 2000). Additional supportive infrastructure included elements such as food outlets; car parking; shower facilities; security systems with 24-h access; on-call sleep areas; accessibility for students with different abilities; and the humane care of research animals (Association of American Medical Colleges, 2012; Australian Medical Council, 2012; Bin Abdulrahman & Saleh, 2015; Cookson, 2013; Field, 2011; Fogarty et al., 2012; Hurt & Harris, 2005; Kebaetse et al., 2014; Liaison Committee on Medical Education, 2006, 2008, 2020; Norris et al., 2006; Schuster et al., 2020; Smego et al., 2010; Snadden et al., 2011; Strasser et al., 2009; Tesson et al., 2009; University of California Riverside, 2008; Whitcomb, 2009, 2013, 2018; Williams et al., 2008; World Federation for Medical Education, 2000, 2015, 2020; Worley et al., 2019). Ideally, spaces were to be designed to encourage group learning, collaboration, mutual support, and a sense of community, even when the medical school was distributed across several locations (Association of American Medical Colleges, 2012; Hurt & Harris, 2005; Lockyer & Patterson, 2005; Schuster et al., 2020).

### Information and technology resources

Incorporating information and communications technology (ICT); and e-learning principles, practices, and resources were commonplace (Association of American Medical Colleges, 2012; Australian Medical Council, 2012; Bin Abdulrahman & Saleh, 2015; Bonner et al., 2018; Chavez et al., 2012; Cookson, 2013; Drobac & Morse, 2016; Fogarty et al., 2012; Frenk et al., 2010; Hays, 2018; Hays et al., 2003, 2019a, b; Howe et al., 2004; Hurt & Harris, 2005; Kebaetse et al., 2014; Khalil & Kibble, 2014; Lanphear & Strasser, 2008; Lawson et al., 2004; Liaison Committee on Medical Education, 2006, 2008, 2020; Lockyer & Patterson, 2005; Mokone et al., 2014; Penner, 2018; Reis et al., 2009; Smego et al., 2010; Snadden et al., 2011; Strasser & Lanphear, 2008; Strasser et al., 2009; Tesson et al., 2009; University of California Riverside, 2008; Whitcomb, 2018; Williams et al., 2008; World Federation for Medical Education, 2000, 2015, 2020; Worley et al., 2019) and were particularly imperative for geographically distributed models of education and distant collaborations with other organisations (Association of American Medical Colleges, 2012; Bonner et al., 2018; Cookson, 2013; Drobac & Morse, 2016; Eichbaum et al., 2014a; Eichbaum et al., 2015; Fogarty et al., 2012; Frenk et al., 2010; Hurt & Harris, 2005; Kebaetse et al., 2014; Lanphear & Strasser, 2008; Mokone et al., 2014; Penner, 2018; Reis et al., 2009; Smego et al., 2010; Snadden et al., 2011; Strasser & Lanphear, 2008; Strasser et al., 2009; Tesson et al., 2009; Williams et al., 2008; Worley et al., 2019). ICT facilities included a varied combination of computers, internet access, smart phones, tablets, personal digital assistants, audio-visual equipment, videoconferencing facilities, smart boards, educational intranets or virtual learning environments, and clinical simulators including virtual reality (Association of American Medical Colleges, 2012; Bin Abdulrahman & Saleh, 2015; Chavez et al., 2012; Cookson, 2013; Fogarty et al., 2012; Hays, 2018; Hays et al., 2003, 2019a, b; Howe et al., 2004; Hurt & Harris, 2005; Kebaetse et al., 2014; Lanphear & Strasser, 2008; Lawson et al., 2004; Liaison Committee on Medical Education, 2008; Lockyer & Patterson, 2005; Smego et al., 2010; Snadden et al., 2011; Strasser & Lanphear, 2008; Strasser et al., 2009; Tesson et al., 2009; University of California Riverside, 2008; World Federation for Medical Education, 2000, 2015, 2020; Worley et al., 2019).

Even though accessing digital information did not necessarily require sophisticated technology, implementing ICT in a new medical school could become quite expensive and logistically complex, which could exacerbate inequities in medical education locally and globally (Drobac & Morse, 2016; Frenk et al., 2010; Hays et al., 2019a, b; Kebaetse et al., 2014; Snadden et al., 2011). Yet, the benefits of new technologies sometimes resulted in their uptake being “faster and more widespread in poor than in rich countries” (Frenk et al., 2010, p. 1945) with technology being a solution for shortages of other kinds of resources including staff (Hays, 2018; Kebaetse et al., 2014; Snadden et al., 2011).

### Accreditation

Accreditation standards were used to assess whether the medical school and its program were sufficient to graduate doctors with the required competencies; and covered aspects such as governance, curriculum content, program delivery, clinical exposure, student selection, student support, and physical space (Australian Medical Council, 2012; Field, 2011; Frenk et al., 2010; Hays, 2018; Liaison Committee on Medical Education, 2006, 2008, 2020; Whitcomb, 2013; World Federation for Medical Education, 2000, 2015, 2020). Not all countries had systems for accreditation, and even when they existed, there were great disparities of quality resulting in many calls for global reform with national and international standardisation (Bin Abdulrahman & Saleh, 2015; Frenk et al., 2010; Howe et al., 2004; Karle, 2010; Mokone et al., 2014; Rizwan et al., 2018; World Federation for Medical Education, 2000, 2015, 2020).

While accreditation standards were not overly prescriptive and left scope for different methods of attainment (Hays, 2018; Whitcomb, 2013), the intense pressure for acceptance caused many new medical schools to make conventional, “safe” choices leading to very similar-looking programs to the detriment of innovation (Hays, 2018, p. 1). Accrediting bodies were cautious to approve educational models and innovations they had never encountered before (Castelo-Branco et al., 2016; Hurt & Harris, 2005; Smith, 2009) making it essential for the new medical school to provide sufficient evidence internally and from international research and examples that the intent of the standard could still be met (Castelo-Branco et al., 2016; Hays et al., 2019a, b; Penner, 2018).

Accreditation was usually a costly and stressful process requiring lengthy, resource-intensive preparation by the new medical school, which may only have a small cohort of staff who were also managing other establishment priorities (Association of American Medical Colleges, 2012; Field, 2011; Hurt & Harris, 2005; Karle, 2010; Smego et al., 2010; Snadden et al., 2011; Whitcomb, 2009, 2013; World Federation for Medical Education, 2000). Common reasons for new medical schools to fail accreditation included limited availability of appropriate academic staff; inadequate access to clinical environments; insufficient financial, physical, and research resources; poor post-graduate employment and training opportunities; lack of realistic forward planning; non-traditional models; short preparation times; and unsatisfactory documentation (Field, 2011; Hurt & Harris, 2005; Whitcomb, 2009, 2013, 2018).

## Discussion

We undertook this scoping review to identify the key factors to be considered when establishing a new medical school, to map the nature of the available literature, and to address the lack of previously published reviews on this topic. Our findings highlight that the evidence-base for the process of new medical school establishment is mainly descriptive in nature, outlining personal and institutional experiences without report of research methodologies nor underpinning theoretical frameworks. Despite the lack of empirical and theoretical foundations to the literature, it could be argued that there is still substantial relevance and utility in experience-based evidence for medical education initiatives (Eva, 2009; Harden et al., 1999). Accreditation guidelines prescribed ‘what’ standards needed to be met without necessarily describing ‘how’ to meet those standards (Australian Medical Council, 2012; Liaison Committee on Medical Education, 2020; World Federation for Medical Education, 2020). Advisory articles and reports, on the other hand, offered practical suggestions and strategic tips on ‘how’ to go about establishing a new medical school without necessarily covering all the aspects of ‘what’ was required (Cookson, 2013; Hays et al., 2019a, b; Snadden et al., 2011). By acknowledging the practical relevance and utility of this experience-based literature, we thematically identified thirteen key considerations that could assist future founding leaders of new medical schools. We summarise the take-home elements of each consideration next.

Whilst the reasons for establishing a new medical school might include improvements to health, services, education, infrastructure, and communities, the almost ubiquitous motivation of workforce shortages/maldistributions indicates how imperative this socio-political driver is. Future founding teams could consider possible location choices accordingly. Appointing tenacious, courageous, committed, and visionary leaders to lead and govern the establishment process will be crucial. The challenge of high costs must be noted and multiple sources plumbed to procure sufficient funds. Partnering in collegial collaborations to share resources could offset some of the high costs. Recruiting the right calibre of staff and retaining them is a common challenge faced by new medical schools, so should be prioritised. Accounting for contextual needs and available resources could help steer the new medical school’s aims for its class sizes. Recruiting the right students through appropriate processes will be crucial in accomplishing the mission and vision of the new medical school. Similarly, curriculum decisions should align with the vision and mission to produce graduates with the desired attitudes and competencies. Affiliations with a wide range of clinical training sites can provide high quality learning experiences and patient encounters. Physical facilities and equipment can range from rudimentary to very sophisticated and founding leaders will need to identify where their needs and abilities intersect. Investment in information and technology resources will be particularly important for geographically distributed models, collaborations with distant stakeholders, and as a solution for shortages of other resources such as staff. Gaining accreditation will be a challenging resource-intensive requirement but can help the founding team significantly improve their approaches and processes.

Even while addressing a significant gap in the literature, our review identified two further gaps. (1) Most authors described their founding efforts following the granting of approvals to proceed with establishment, with minimal discussion of how to go about obtaining the initial green-light from governing authorities. For visionary leaders seeking to establish a new medical school, understanding how to successfully obtain this permission from governments, universities, health systems, and accrediting bodies, would be essential. (2) There was minimal exploration of the personal costs and burnout experienced by founding leaders and staff of new medical schools. High staff turnover rates were noted by some new medical schools (Mokone et al., 2014; Nausheen et al., 2018; Worley et al., 2019), but impacts of stress and burnout were not highlighted. Given the crucial nature of good staffing for the successful establishment of a new medical school, a better understanding of these potential challenges would be paramount.

### Strengths, limitations, and areas for future research

A scoping review was ideal for analysing this broad, complex, and heterogenous topic, especially given the dearth of prior research, published literature reviews, and applied theories. However, the iterative refinement of inclusion and exclusion criteria based on ‘best fit’ in scoping reviews can lend itself to interpretive subjectivity. The unlimited date ranges and publication types facilitated broad coverage of a poorly studied topic, but too large a scope could limit the analysis to a superficial treatment and/or a lengthy treatise. Although limiting the articles to English-language provided some level of bounding, it may have narrowed understanding and missed key multi-cultural perspectives.

Addressing the dearth of research and theoretical foundations for the process of new medical school establishment identified through this scoping review, we undertook retrospective, international Critical Realist Multiple Case Study and applied Institutional Entrepreneurship theory borrowed from the business domain (Kirubakaran, 2022; Kirubakaran et al., 2024). Future research could prospectively apply and critique our findings. Future research could also consider using different research methodologies such as Participatory Action Research or Impact Evaluation, and applying different theoretical frameworks such as Diffusion of Innovations theory or Change Management theory.

## Conclusions

This inaugural scoping review on the *process* of new medical school establishment addresses a major gap in the literature. Although there is a paucity of research and theory underpinning existing publications, the available descriptive and experience-based evidence is still useful and reveals thirteen key factors for leaders and founding teams to consider. There is still, however, need for more theoretically and empirically informed research on this significant and complex undertaking to assist future founding leaders and teams to maximise the outcomes and impact of their establishment efforts.

## Supplementary Information

Below is the link to the electronic supplementary material.

Supplementary file1 (DOCX 0 KB)

## Data Availability

No datasets were generated or analysed during the current study.
