# Supplementary figures and images for: How to establish a new medical school? A scoping review of the key considerations
Source: Adv Health Sci Educ Theory Pract. 2024 Sep 4;30(2):645–69. doi: 10.1007/s10459-024-10370-y (PMC11965218; doi:10.1007/s10459-024-10370-y)

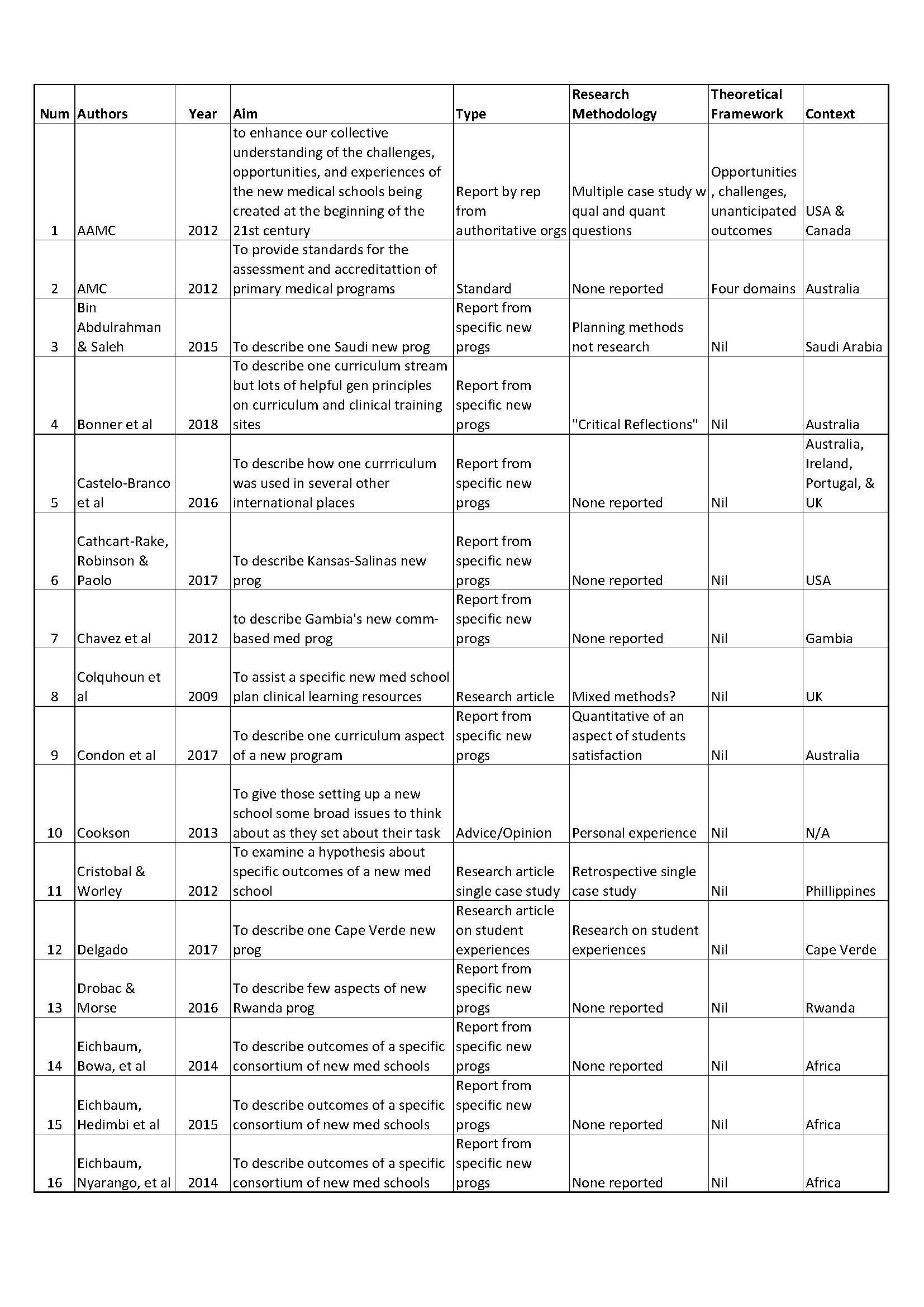
Supplemental Digital Appendix: Charting the data


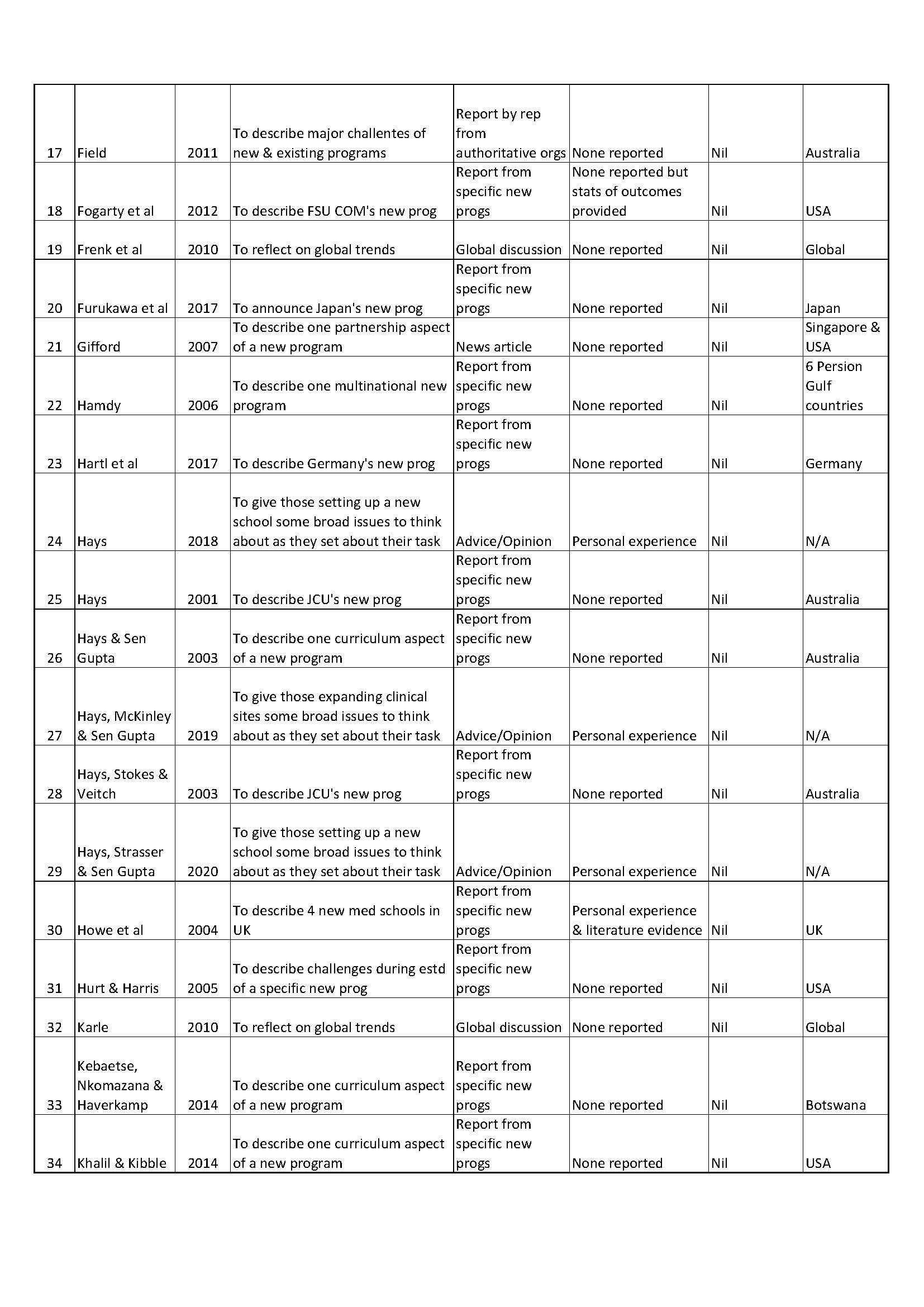


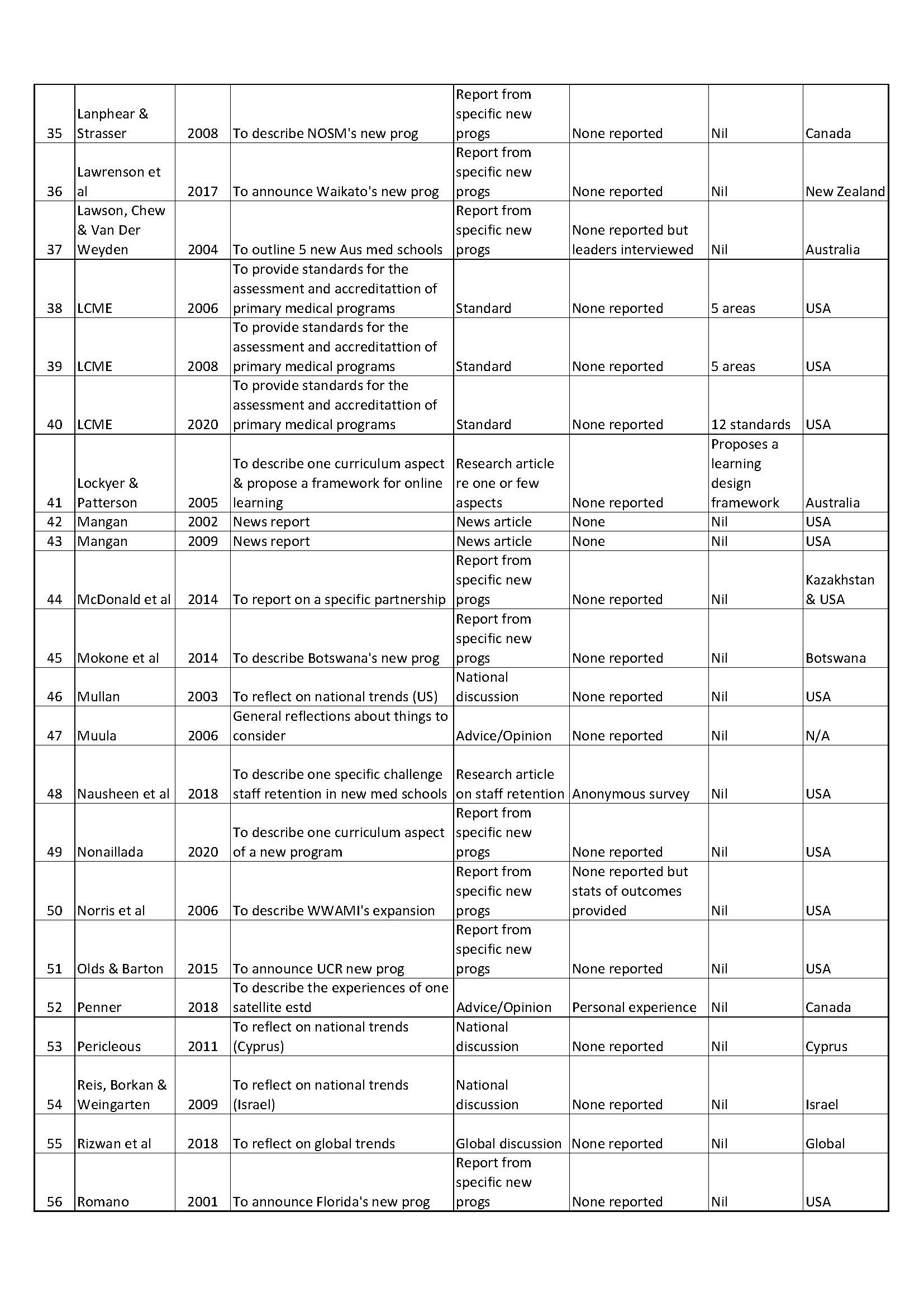


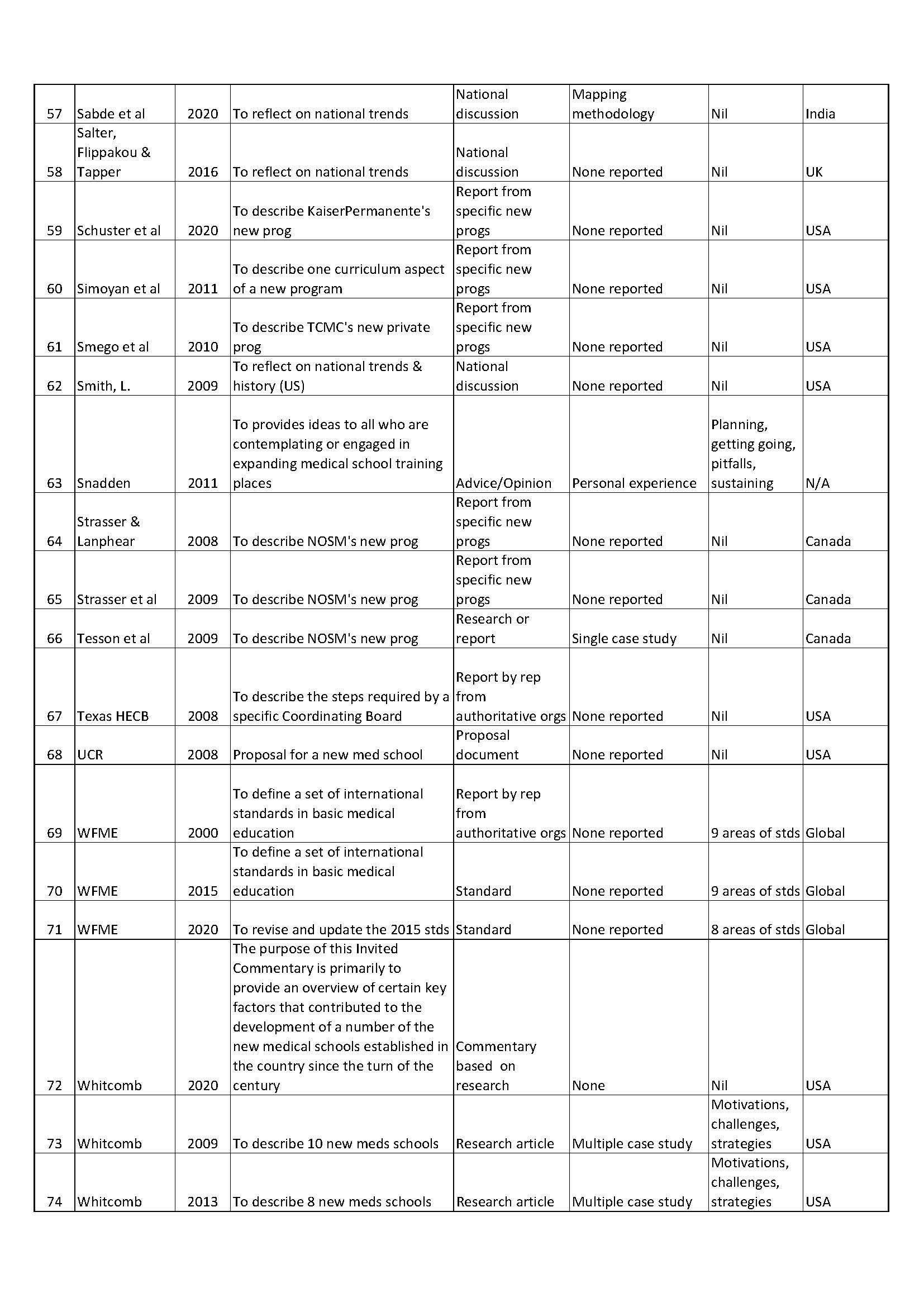


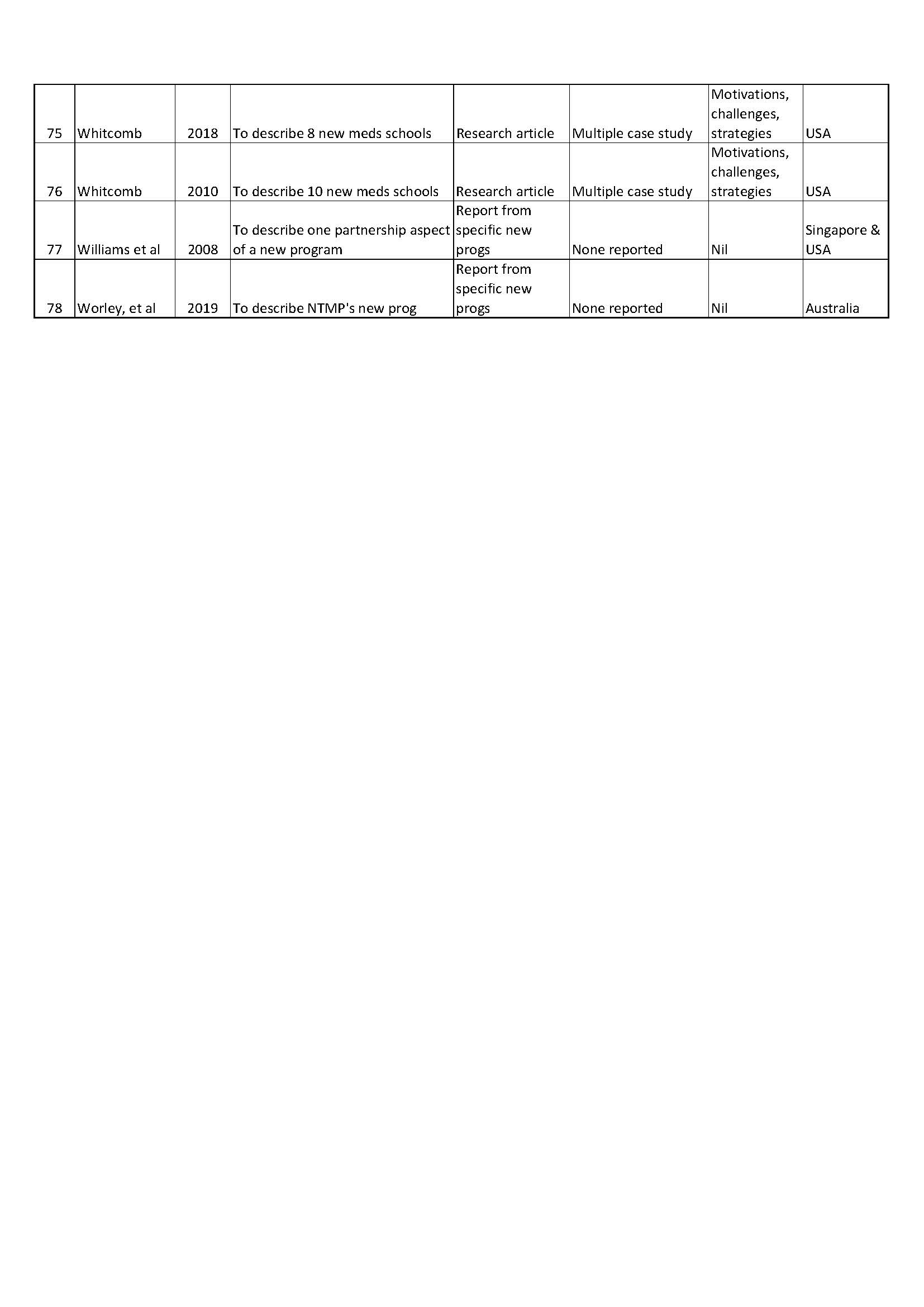

Supplement: Supplementary file 1 — Supplementary file1 (DOCX 0 KB) [file 10459_2024_10370_MOESM1_ESM.docx]
